# Extended Multi WLS Method for Lossless Image Coding

**DOI:** 10.3390/e22090919

**Published:** 2020-08-22

**Authors:** Grzegorz Ulacha, Ryszard Stasiński, Cezary Wernik

**Affiliations:** 1 Faculty of Computer Science and Information Technology, West Pomeranian University of Technology, ul. Żołnierska 49, 71-210 Szczecin, Poland; cwernik@wi.zut.edu.pl; 2 Department of Informatics and Telecommunications, Poznań University of Technology, ul. Piotrowo 3, 60-965 Poznań, Poland; ryszard.stasinski@put.poznan.pl

**Keywords:** image coding, lossless coding, arithmetical coder

## Abstract

In this paper, the most efficient (from data compaction point of view) and current image lossless coding method is presented. Being computationally complex, the algorithm is still more time efficient than its main competitors. The presented cascaded method is based on the Weighted Least Square (WLS) technique, with many improvements introduced, e.g., its main stage is followed by a two-step NLMS predictor ended with Context-Dependent Constant Component Removing. The prediction error is coded by a highly efficient binary context arithmetic coder. The performance of the new algorithm is compared to that of other coders for a set of widely used benchmark images.

## 1. Introduction

Image compression methods can be divided into lossy and lossless. Important applications of lossless image and video compression techniques include archiving of 2D, 3D, and 4D (3D video sequences) medical images [1,2,3,4,5], as well as astronomical and satellite ones [6,7]. Lossless mode is often required at some stage of photographs, advertising materials, TV productions, and films graphic processing (post-production [8]), etc. In such cases, lossy versions of compression methods, such as JPEG, JPEG2000 (for static images) [9], MPEG2, and MPEG4 [10] (for video sequences), cannot be used. Although these standards have appropriate lossless modes, they are not particularly efficient.

Samples of multimedia signals are usually strongly correlated, which means that simple entropy coders do not compress them effectively. Correlation can be significantly reduced by signal prediction, but then another problem emerges: changes in signal statistical properties. In the 1990s, several solutions were proposed that used linear and non-linear prediction for lossless image compression. The first image coder that solved the above problems relatively well was the context coder CALIC [11]. At that time, it was considered too complex for practical purposes; nevertheless, the standardized JPEG-LS was a context coder too [12]. Then followed more efficient but also more complex techniques: TMW^*Lego*^ [13], Multi-WLS [14], MRP 0.5 [15]. The more recent best algorithms are Blend-20 [16], and then the improved versions of MRP 0.5: GPR-BP [17], and MRP-SSP [18]. Further analysis of currently existing solutions is presented in Section 1.2.

In this paper, the currently best image lossless coding algorithm is presented, extended multi weighted least squares (EM-WLS; Section 3.2), and its simplified version, locally adaptive ordinary least squares (LA-OLS; Section 3.1). The algorithms have in common the cascade structure proposed in Section 2; their first stages are either EM-WLS, or LA-OLS predictors. Section 6.4 shows that it is much better than any older method, including the newest GPR-BP, and MRP-SSP. EM-WLS is an extended version of the WLS technique, which is also a basis for the very good multi-WLS algorithm [14]. The first version of AVE-WLS was introduced [19]; the current version using the cascade prediction system has been significantly improved (Section 2). Several new formulas are added, i.e., additional NLMS stages (Section 3.3), enhanced cumulated prediction error cancellation stage (Section 4), and a new arithmetic coder (Section 5).

### 1.1. Basics of Prediction Coding

Optimization of data encoding consists of minimizing the average number of bits per single symbol generated by a source S (in the case of lossless, image compression symbols are pixel color values; in this paper, we work with 8-bit luminance).

**Figure 1:**
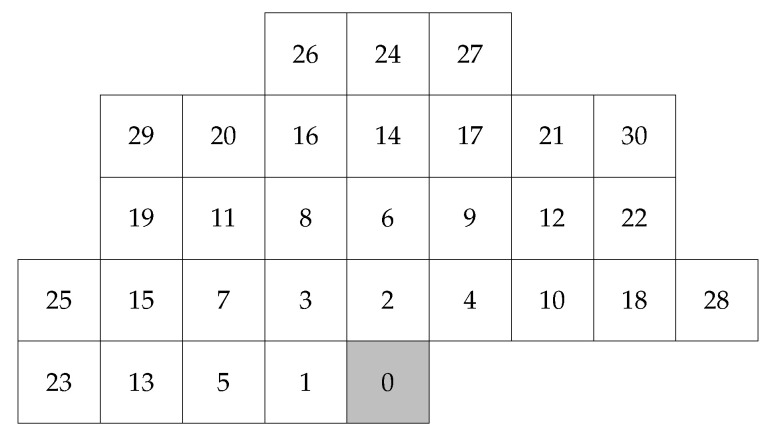
Numbering of neighborhood pixels or errors of P(0) or e(0).

Depending on the sources, we can divide them into those without memory (discrete memoryless source (DMS)) and sources with memory (conditional source model (CSM)) [20]. Considering this classification from the Markov model point of view, in the first case, the lower limit on the bit average is the unconditional entropy ($H(S_{\mathrm{DMS}})$, also named zero-order entropy). In the second case, we deal with the conditional entropy of the kth order, defined as $H(S|C^{\left(k\right)})$, where in the case of images context, $C^{\left(k\right)}$ may be defined, e.g., by k neighbor pixels (Figure 1).

In general, for the source entropy H(S), the following relation holds: $H\left(S\right)\leq H\left(S|C^{\left(k\right)}\right)\leq H\left(S_{\mathrm{DMS}}\right)$. Since image samples take on many values (at least 256), it is impractical to determine and apply a Markov model for them. It is usually assumed that removal of inter-dependencies between pixels is possible by using predictive techniques; prediction errors are coded. A linear predictor of order r is used to estimate the value of a sample:

(1) $$\hat{x}\left(0\right)=\sum_{i=1}^{r}w(i)\cdot P(i),$$

where P(i) is pixel from the currently coded pixel x(0)=P(0) neighborhood (Figure 1), and w(i) is a predictor coefficient from vector w=[w(1),w(2),…,w(r)] [21]. To simplify notation in this work, indices of the currently coded pixel are usually omitted, while indices i of pixels P(i) and prediction errors e(i) show their distances from the processed element P(0) or e(0) (Figure 1):

The closest pixels provide the most information about the coded one, so the neighboring pixels are ordered in accordance with their Euclidean distances from P(0) (Figure 1). The numbering of pixels with the same distance from P(0) is usually determined clockwise (this is discussed in more detail in Section 3.2.3). The prediction model can be a linear model of the kth order or a more complex nonlinear solution, but we reduce it to a predictor of one value $\hat{x}\left(0\right)$, which is then subtracted from the current P(0), see Formula (2). In this way, we create a not explicitly defined first-order Markov model. As a result, a sequence of prediction errors can be expected to form a source for which the first-order entropy value is noticeably smaller than the zero-order value. The estimated pixel value (expected value rounded to the closest integer) is subtracted from the real pixel value:

(2) $$e\left(0\right)=x\left(0\right)-\left\lfloor \hat{x}\left(0\right)-0.5\right\rfloor ,$$

and the difference (prediction error e(0)) coded. In this way, we obtain a differential image, in which probability distribution of its samples is close to the Laplace distribution [21,22]. This allows efficient coding of prediction errors using one of the static or adaptive entropy methods, among which arithmetic coding is most effective (Section 5).

Notably, it is extremely difficult to determine image entropy H(S) because it is hard to define a Markov model that considers all interdependencies between pixels. Namely, the dependencies may extend quite far. An example is the research use of linear prediction of order $r_{2}=106$ in the second stage of the proposed-here cascade structure (Section 3.3). The number of Markov model states grows exponentially with its order. Here, if we assume that symbols are 8-bit pixel values, the number is at least $256^{106}$. Therefore, only the bit average, being the average number of bits necessary for coding a pixel, is used as the basic benchmark for testing lossless codecs (Section 6).

### 1.2. Adaptive Predictive Techniques

The effectiveness of image compression depends on the correct choice of one or more prediction models. Linear prediction is most often used (Formula (1)), where a vector w containing r predictor coefficients w(i) is usually determined by minimizing the mean square error (MMSE). This vector can be determined once for the whole image by a forward adaptation method or individually for each coded pixel using a backward adaptation technique. The advantage of backward adaptation is the possibility of using relatively high prediction orders (there is no need to provide prediction coefficients to the decoder), which allows for high compression efficiency. However, the disadvantage of this solution is the necessity of updating prediction coefficients in both the encoder and decoder for each pixel, which is a time-symmetric approach.

In methods with forward adaptation, it is reasonable to divide images into blocks (e.g., 8×8 or 16×16 pixels) and define an individual predictive model for each. One of the first solutions of this type was the method presented by Memon [23], where one of eight fixed models was assigned to each block of 8×8 pixels, producing the smallest absolute error. As such, the header information associated with a block required only three bits, forming the model number.

Subsequent methods used the mean square error for determining the best prediction coefficients. Unfortunately, this was associated with very large header information, as predictor coefficients required large numbers of bits. To reduce the size of the header, the blocks with similar characteristics were grouped into clusters, and all were associated with a common prediction model [24]. Through using vector quantization techniques (as well as fuzzy clustering [25]), optimized sets of, e.g., 16 prediction models were created, so that even for a large prediction order, the header size did not significantly increase the bit average. Matsuda et al. [15] used a technique of combining adjacent blocks belonging to the same category into groups (associated with the same predictor) to create larger blocks. Next, a map of the blocks with variable sizes was saved using an effective technique of encoding quadtrees.

In the above-mentioned above block techniques, it is easy to prove that it is possible to reach lower values of first-order entropy than for the MMSE method [15]. In the paper [26], it was proposed to replace MMSE criterion with minimum mean absolute error (MMAE), which improved the results for a block method. The poblem of discrepancy between MMSE and first-order entropy criterions was discussed in paper [27].

In the case of backward adaptation in local training windows, the MMSE criterion is closer to optimal one than for forward adaptation approaches. Analysis of this issue is presented in Section 6.1. This type of solution was used in the lossless image codec proposed in this work.

## 2. Cascade Prediction Model

Considering the features of adaptive methods described in Section 1.2, to achieve the highest possible compression efficiency in our work, we decided to use a backward adaptation approach, and developed a highly effective cascade prediction model whose final high-efficiency form is discussed in this section. A similar approach was used earlier in lossless audio compression solutions [28]. The cascade is calculated as follows: In the main stage image, pixels are processed:

(3) $$y_{1}\left(0\right)=\sum_{i=1}^{r_{1}}w_{MP}\left(i\right)\cdot P(i),$$

where coefficients of the predictor form vector $w_{MP}$ of order $r_{1}$. They are computed using the WLS or OLS methods described in Section 3.1 and Section 3.2, respectively. In the following stages, prediction errors from previous stages are transformed:

(4) $$y_{j}\left(0\right)=\sum_{i=1}^{r_{j}}w_{j}\left(i\right)\cdot e_{j-1}\left(i\right),\;\mathrm{for}\;j>1,$$

(5) $$e_{1}\left(0\right)=P\left(0\right)-y_{1}\left(0\right),$$

(6) $$e_{j}\left(0\right)=e_{j-1}\left(0\right)-y_{j}\left(0\right),\;\mathrm{for}\;j>1.$$

**Figure 2:**
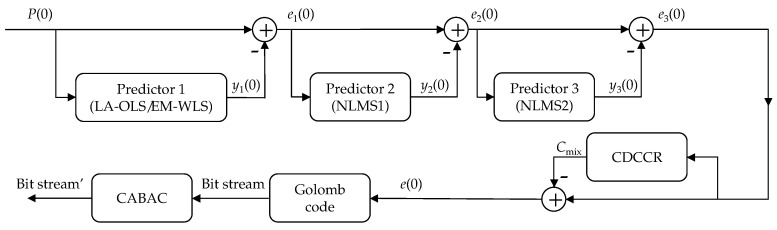
Cascade of predictors forming the data modelling part of the coder.

The final prediction error is obtained at the output of a cascade structure shown in Figure 2. It is given by:

(7) $$e\left(0\right)=P\left(0\right)-\left\lfloor y_{1}\left(0\right)+y_{2}\left(0\right)+y_{3}\left(0\right)+C_{\mathrm{mix}}+0.5\right\rfloor ,$$

where $y_{1}\left(0\right)$ and $y_{2/3}\left(0\right)$ are signal estimates provided by the Main Predictor, the locally adaptive Ordinary Least-Squares (OLS) method (LA-OLS, Section 3.1) or the extended multi WLS method (EM-WLS, Section 3.2), and then by Normalized Least Mean Square predictors (NLMS+, Section 3.3) respectively. Finally, $C_{\mathrm{mix}}$ is the cumulated prediction error correction defined in Section 4 (Context-Dependent Constant Component Removing (CDCCR)). The last two blocks (Golomb code and Context Adaptive Binary Arithmetic Coder (CABAC)) are used for effective compression of predictive errors. Details are provided in Section 5.

The proposed solution can be considered to be a model offering the highest efficiency. To reduce implementation complexity (Section 6.4), individual blocks can be easily modified (e.g., by reducing prediction orders in the first 3 blocks) or even deleted. For example, block Predictor 1 may contain one of two versions of the Main Predictor: EM-WLS or the faster LA-OLS. The blocks are described in the following sections, and the effects of blocks removal are discussed in more detail in Section 6.

## 3. Stages of Adaptive Predictive Cascade

In this section, we present the blocks in the first three stages of the cascade structure from Figure 2. In particular, two versions of the Main Predictor are defined: complex but more efficient EM-WLS and simplified LA-OLS.

### 3.1. Locally Adaptive OLS Method

In Section 1.2, we list examples of codecs using predictive methods with forward adaptation. Among them, the most effective are those that adapt the prediction model to local features of an image, e.g., for 8×8 or 16×16 pixel blocks. In the case of backward adaptation, similarly sized *Q* windows are used. The main difference is that the currently encoded pixel P(0) is not in that *Q* windows.

**Figure 3:**
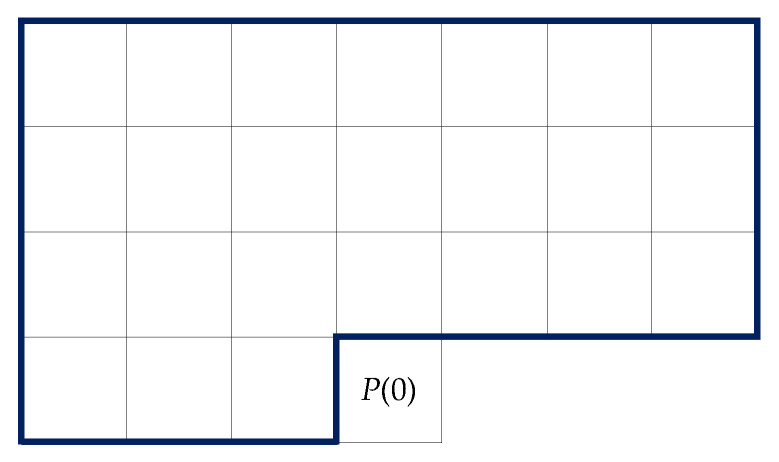
Training window *Q* for window parameter W=3.

In the OLS method, prediction coefficients are calculated for each coded pixel, minimizing the prediction mean square error in a certain limited area *Q*. The vector of OLS predictor coefficients $w_{MP}$ (Main Predictor) is computed from the following formula [29]:

(8) $$w_{MP}=R^{-1}\cdot P,$$

where R is the experimental signal autocovariance matrix:

(9) $$R\left(j,i\right)=\sum_{y\in Q}\sum_{x\in Q}\Psi_{\left(y,x\right)}\cdot P_{\left(y,x\right)}\left(i\right)\cdot P_{\left(y,x\right)}\left(j\right),$$

and vector P is:

(10) $$P\left(j\right)=\sum_{y\in Q}\sum_{x\in Q}\Psi_{\left(y,x\right)}\cdot P_{\left(y,x\right)}\left(0\right)\cdot P_{\left(y,x\right)}\left(j\right),$$

where Ψ is a weighting function, which takes a constant value of 1 for classic OLS; pixels *P* are taken from a training window *Q* around the coded pixel located at position (y,x) (Figure 3). It consists of *W* image sub-rows of size 2W+1 preceding the estimated pixel and *W* pixels preceding it in the current row (*Q* is an area extending *W* pixels to the left and up from the coded pixel P(0) and to the right in rows preceding it). The best results were obtained for W=10,r=18 (Section 6.2).

The default vector $w_{MP}=\left[0.620,0.625,-0.125,0.125,-0.125,-0.125\right]$ is used in border areas when R is ill-conditioned. With the *Q* training window defined in this way and coding of successive pixels row by row using a fast sliding window procedure, the adaptation of the R matrix and vector P is performed relatively fast in comparison to the WLS method discussed in Section 3.2. It involves deleting data obtained from the extreme left column of the *Q* area and including the new extreme right column of the training window [30].

In the paper, the improved version of this scheme is analyzed. Firstly, a more robust version of Formula (8) is used [31,32]:

(11) $$w_{MP}={\left(R+u_{\mathrm{bias}}\cdot I\right)}^{-1}\cdot P,$$

where $u_{\mathrm{bias}}=800$ (term $u_{\mathrm{bias}}I$ guarantees non-singularity of (9)).

Secondly, predictor coefficients obtained from (11) are weighted by coefficients:

(12) $$\bar{w}\left(j\right)=w\left(j\right)\cdot \frac{\sqrt[{4}]{{\bar{d}}_{j}}}{\sum_{i=1}^{r}\sqrt[{4}]{{\bar{d}}_{i}}},$$

where ${\bar{d}}_{j}=1/\sqrt{{\left(\Delta x_{j}\right)}^{2}+{\left(\Delta y_{j}\right)}^{2}}$ is the inverse Euclidean distance from the currently coded pixel. The new coefficients substitute w(j) in (3), which is a novel idea introduced in this algorithm.

Another improvement proposed in this work is the introduction of a weighting function promoting pixels, for which prediction errors at their positions in window *Q* are small:

(13) $$\Psi_{\left(y,x\right)}=\frac{1}{4+|e_{\left(y,x\right)}\left(0\right)|}.$$

In this case, $u_{\mathrm{bias}}=100$. As such, the weighted sum $\sum_{y\in Q}\sum_{x\in Q}\Psi_{\left(y,x\right)}\cdot e_{\left(y,x\right)}^{2}\left(0\right)$ of squared errors is minimized, similar to the WLS method described in Section 3.2.

### 3.2. Multi WLS Method

The use of a more complex weighting function compared to Formula (13)) requires a significant increase in implementation complexity at the stage of determining the R matrix and P vector. EM-WLS is based on the WLS concept, so this section begins with its description.

#### 3.2.1. Weighted Least-Squares (WLS)

**Figure 4:**
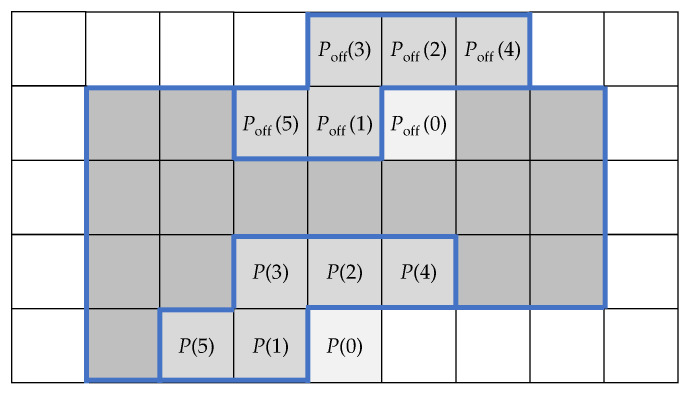
Neighborhoods of pixels P(0), and $P_{\mathrm{off}}\left(0\right)$ for m=5 and W=3.

In general, the vector of WLS predictor coefficients $w_{MP}$ is computed from Formulas (9)–(11) [21]. The weighting function Ψ reflects similarity between neighborhoods of size *m* around the coded P(0) and some other $P_{\mathrm{off}}\left(0\right)$ pixels [14,19] (Figure 4). The initial form of the function was relatively simple [14]:

(14) $$\Psi =\frac{1}{1+\sum_{k=1}^{m}{\left(P\left(k\right)-P_{\mathrm{off}}\left(k\right)\right)}^{2}}.$$

#### 3.2.2. AVE-WLS Method

A previous paper on AVE-WLS [19] followed suggestions from [33,34], as did we, concerning the optimal form of LS predictors. The first one was a statement that for each coded pixel, an optimal predictor order exists [33]. It was not known a priori, so in [19], instead of searching for it, we propose computing the averaged value of the WLS predictor vectors $w_{MP(j)}$ for orders from $j=r_{\min}$ to $j=r_{\max}$:

(15) $$w_{\mathrm{AVE}-\mathrm{MP}}=\frac{1}{r_{\max}-r_{\min}+1}\cdot \sum_{j=r_{\min}}^{r_{\max}}w_{MP(j)}.$$

Implemented here, the predictor orders range from $r_{\min}=4$ to $r_{\max}=24$. Additionally, W=14 (Figure 3). If vectors $w_{MP(j)}$ should be extended to $r_{\max}$, it is achieved by zero-padding.

In this paper, a complex weighting function is proposed. Equation (16) is an enhanced version of the formula from [19]:

(16) $$\Psi_{\left(y,x\right)}=\alpha \cdot \frac{\lambda_{2}+0.8^{\sqrt{{\left(\Delta y_{0}\right)}^{2}+{\left(\Delta x_{0}\right)}^{2}}}}{{\left(\lambda_{1}+\sum_{k=1}^{m}{\left({\bar{d}}_{k}\cdot \left(P\left(k\right)-P_{\mathrm{off}}\left(k\right)\right)\right)}^{2}\right)}^{\gamma }},$$

where $\lambda_{1}=64$, $\lambda_{2}=0.25$, ${\bar{d}}_{k}=1/\sqrt{{\left(\Delta y_{k}\right)}^{2}+{\left(\Delta x_{k}\right)}^{2}}$ is the inverse of Euclidean distance between pixels P(k) and P(0), neighborhood size $m=r_{\max}$ (Formula (15)), and γ=1.18. The expression appearing as a power of number 0.8 determines the Euclidean distance between pixels P(0) and $P_{\mathrm{off}}\left(0\right)$. The scaling factor α depends on two threshold values calculated by Algorithm 1:
**Algorithm 1:** Algorithm for calculating α weight (Formula (16))
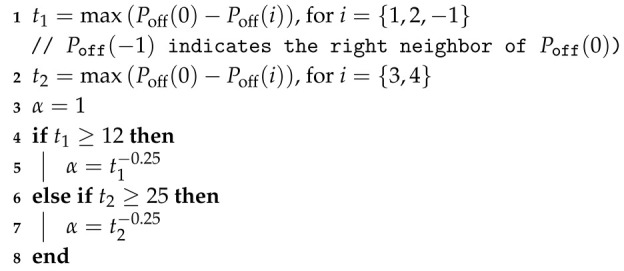


The second suggestion concerning LS predictors optimization [34] was a proposition to use not all, but only $r_{\max}$ most similar (correlated) pixels taken from a range of $r_{\mathrm{ext}}$ pixels, $r_{\mathrm{ext}}>r_{\max}$, in the basic prediction Formula (1). In this paper, we propose to set $r_{\max}=24$ and $r_{\mathrm{ext}}=48$, but similarity is tested using only 10 pixels with indices from 15 to 48, and located in *Q* (Figure 4). The similarity measure here is the smallest cumulative absolute distance of pixels from the Q region:

(17) $$\rho_{\mathrm{dist}}\left(k\right)=\sum_{y\in Q}\sum_{x\in Q}\left|P_{\left(y,x\right)}\left(0\right)-P_{\left(y,x\right)}\left(k\right)\right|.$$

Apart from the chosen 10 pixels, the remaining ones are the closest to the coded one. The minimization rule (17) does not apply to the 14 nearest pixels; they are considered by default. In this way, vector $Z=\{z\left(1\right),z\left(2\right),...,z\left(r_{\max}\right)\}$ is calculated from $r_{\max}=24$ pixels P(i) from the neighborhood of size $r_{\mathrm{ext}}=48$. Formula (3) takes the form:

(18) $$y_{1}\left(0\right)=\sum_{i=1}^{r_{\max}}w_{\mathrm{AVE}-\mathrm{MP}}\left(i\right)\cdot z\left(i\right).$$

Finally, $u_{\mathrm{bias}}$ is also optimized in this paper using ridge regression [35]. Firstly, the initial vector $w_{\mathrm{AVE}-\mathrm{MP}}$ is calculated for $u_{\mathrm{bias}}=0$, then the following term is computed:

(19) $$u_{\mathrm{bias}}=c\cdot \frac{\sum_{y\in Q}\sum_{x\in Q}\Psi_{\left(y,x\right)}\cdot e_{\left(y,x\right)}^{2}\left(0\right)}{\sum_{i=1}^{r_{\max}}{\left(w_{\mathrm{AVE}-\mathrm{MP}}\left(i\right)\right)}^{2}},$$

where e(0) is defined in (2), respectively; $w_{\mathrm{AVE}-\mathrm{MP}}\left(i\right)$ is the coefficients of the mentioned above initial vector $w_{\mathrm{AVE}-\mathrm{MP}}$. Constant c can be evaluated as $4\cdot r_{\max}/W^{2}\approx 0.5$.

#### 3.2.3. Extended Multi WLS Method

Another idea introduced in this work, apart from the proper selection of the neighborhood (Section 6.3), is replacement of the arithmetic mean of 22 prediction models for successive orders from $r_{\min}=3$ to $r_{\max}=24$ (Formula (15)) by a weighted mean whose weights are determined adaptively after coding of each pixel using an improved ALCM+ method. The approach of weighted combination of predictive models (for calculation of the Main Predictor) is called the extended multi WLS method (EM-WLS).

The original version of the Activity Level Classification Model (ALCM) technique [36] was developed in the 1990s for image coding purposes. In comparison to the classic LMS solution, it is characterized by a lower, though similar, computational complexity. Originally, the method operated on linear predictors of the fifth or sixth order. In each iteration, only two predictor coefficients were modified, and only by adding/subtracting a constant (μ=1/256 for 8-bit samples). Here, the number of coefficients is 22, and up to six properly selected coefficients are modified, hence the name ALCM+.

In the proposed solution, Formula (15) is extended to the following:

(20) $$w_{\mathrm{ALCM}-\mathrm{MP}}=\sum_{j=r_{\min}}^{r_{\max}}\beta_{j}\cdot w_{\mathrm{MP}(j)},$$

where weights $\beta_{i}$ are initialized with $1/(r_{\min}-r_{\max}+1)$, so the sum of weights is 1. Formula (18) takes the form:

(21) $$y_{1}\left(0\right)=\sum_{i=1}^{r_{\max}}w_{\mathrm{ALCM}-\mathrm{MP}}\left(i\right)\cdot z\left(i\right).$$

To determine the updated $\beta_{j}$, it is necessary to calculate predictions ${\hat{x}}_{MP(j)}\left(0\right)$ given by WLS models for orders of predictors from $r_{\min}=3$ to $r_{\max}=24$. A 22-element vector $g=\{{\hat{x}}_{MP(j)}\left(0\right)\}$ is created:

(22) $${\hat{x}}_{MP(j)}\left(0\right)=\sum_{i=1}^{r_{j}}w_{MP(j)}\left(i\right)\cdot z\left(i\right),\;\mathrm{for}\;j=\left\{1,2,...,22\right\},r_{j}=\left\{3,4,...,24\right\}.$$

After coding the current pixel P(0), six weights $\beta_{j}$ are adapted. For this purpose, the highest three and the lowest values three are taken from the vector g. Let us denote these elements as the smallest:

(23) $$g\left(q_{\left(1\right)}\right)\leq g\left(q_{\left(2\right)}\right)\leq g\left(q_{\left(3\right)}\right),$$

and the largest:

(24) $$g\left(p_{\left(3\right)}\right)\leq g\left(p_{\left(2\right)}\right)\leq g\left(p_{\left(1\right)}\right).$$

If condition $g\left(q_{\left(1\right)}\right)<g\left(p_{\left(1\right)}\right)$ is true, then the following adaptation coefficients are used (Algorithm 2):
**Algorithm 2:** Adaptation of weights $\beta_{j}$.

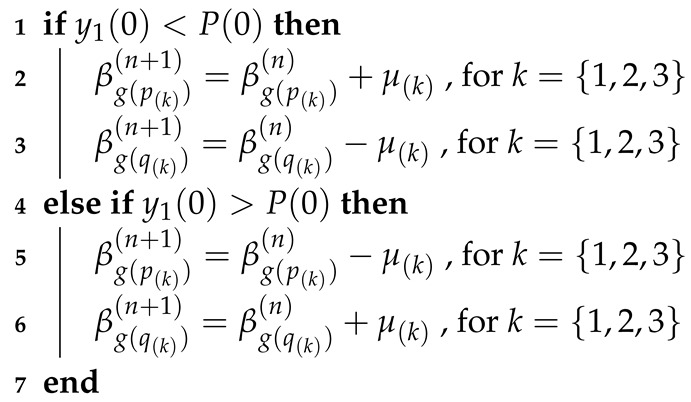



The modifying value $\mu_{\left(k\right)}$ is determined as:

(25) $$\mu_{\left(k\right)}=\min\left\{2^{-12};{\bar{\mu }}_{\left(k\right)}\right\},$$

where:

(26) $${\bar{\mu }}_{\left(k\right)}=\frac{\left|P\left(0\right)-y_{1}\left(0\right)\right|}{2^{6}\cdot \sum_{j=1}^{3}\alpha_{j}\cdot \left(g\left(p_{\left(j\right)}\right)-g\left(q_{\left(j\right)}\right)\right)},$$

and $\alpha_{j}=\left\{1;0.75;0.5\right\}$.

### 3.3. Normalized Least Mean Square (NLMS) Method

Similar to [28,37], in this paper, we use the cascaded NLMS method for tuning the results obtained from the Main Predictor. This approach allows introduction of high prediction orders while maintaining relatively low implementation complexity. Here we have two cascaded NMLS filters with orders $r_{3}<r_{2}$. The values of prediction coefficients are initially set to 0, i.e., $w_{j}^{\left(n=0\right)}=\left[0,...,0\right]$, where *j* is the prediction stage (for NLMS j={2,3}) of the cascade prediction model shown in Figure 2. The general coefficient update formula for the NLMS method is [21]:

(27) $$w_{j}^{\left(n+1\right)}\left(i\right)=w_{j}^{\left(n\right)}\left(i\right)+\mu_{j}\left(i\right)\cdot {\bar{e}}_{j}^{\left(n\right)}\left(0\right)\cdot e_{j-1}^{\left(n\right)}\left(i\right),\;\mathrm{for}\;i=\left\{1,2,...,r_{j}\right\},$$

where

(28) $${\bar{e}}_{j}^{\left(n\right)}\left(0\right)=\mathrm{sgn}\left(e_{j}^{\left(n\right)}\left(0\right)\right)\cdot \min\left\{\left|e_{j}^{\left(n\right)}\left(0\right)\right|;\varphi \right\},$$

is the bounded prediction error. The experimentally found bound φ=14.

In the NLMS approach, the learning coefficient $\mu_{j}\left(i\right)$ adapts to the signal. Here, we propose an enhanced formula for it [31]:

(29) $$\mu_{j}\left(i\right)=\frac{{\bar{d}}_{i}}{2^{3}\cdot \sqrt{{\bar{\sigma }}^{2}}\cdot \left(10+\sum_{k=1}^{r_{j}}\sqrt{{\bar{d}}_{k}}\cdot {\left(e_{j}^{\left(n\right)}\left(k\right)\right)}^{2}\right)},$$

where $e_{j}^{\left(n\right)}\left(k\right)$ is the kth signal sample (Figure 1). Here, the prediction error from the preceding stage of the algorithm, ${\bar{\sigma }}^{2}$, is the weighted average value of all variances ${\bar{\sigma }}^{2}$:

(30) $${\bar{\sigma }}^{2}=\frac{1}{\delta }\sum_{k=1}^{m}{\bar{d}}_{k}\cdot {\left(P\left(k\right)-\bar{p}\right)}^{2},$$

where m=10, $\bar{p}=\frac{1}{\delta }\sum_{k=1}^{m}{\bar{d}}_{k}\cdot P\left(k\right)$ and $\delta =\sum_{k=1}^{m}{\bar{d}}_{k}$.

The orders of NLMS predictors used in this paper are $r_{2}=96$, and $r_{3}=30$ for LA-OLS, and $r_{2}=106$ and $r_{3}=42$ for EM-WLS.

## 4. Cancelling Cumulative Prediction Error

### 4.1. Context-Dependent Constant Component Removal

It was observed for the first time in [11] that predictors tend to produce a DC error component, which diminished their efficiency. Then, the algorithm for computing the correction value for cancelling this component was constructed. According to Formula (7), in our algorithm, the final pixel estimate and its rounded version are:

(31) $$\dot{x}\left(0\right)=y_{1}\left(0\right)+y_{2}\left(0\right)+y_{3}\left(0\right)+C_{\mathrm{mix}},$$

(32) $$\hat{x}\left(0\right)=\left\lfloor \dot{x}\left(0\right)+0.5\right\rfloor ,$$

where the components come from the Main Predictor, NLMS1, NLMS2, and the cumulative error correction stages. The latter is computed by using an extended formula from our previous works [38]:

(33) $$C_{\mathrm{mix}}=\sum_{j=1}^{12}\alpha_{j}\cdot C_{j},j=4\left(f-1\right)+k,$$

where $C_{j}$ components are computed using context-based error bias correction methods mentioned in [38]: there are 4 context definitions for each technique, k={1,2,3,4}; hence, j={1,2,3,4} for JPEG-LS, f=1; j={5,6,7,8} for CALIC, f=2; j={9,10,11,12} for the slightly modified median method, and f=3. For j≤8, $C_{j}$ is obtained from $C_{j}=S_{\mathrm{map}}\left(j\right)/N_{\mathrm{map}}\left(j\right)$, where $S_{\mathrm{map}}\left(j\right)$ are elements of $S_{\mathrm{map}}=[S_{{\mathrm{JPEG}-\mathrm{LS}}_{1}}\left(i_{1}\right),S_{{\mathrm{JPEG}-\mathrm{LS}}_{2}}\left(i_{2}\right),S_{{\mathrm{JPEG}-\mathrm{LS}}_{3}}\left(i_{3}\right),S_{{\mathrm{JPEG}-\mathrm{LS}}_{4}}\left(i_{4}\right),S_{{\mathrm{CALIC}}_{1}}\left(i_{1}\right),S_{{\mathrm{CALIC}}_{2}}\left(i_{2}\right),S_{{\mathrm{CALIC}}_{3}}\left(i_{3}\right),$ $S_{{\mathrm{CALIC}}_{4}}\left(i_{4}\right)]$ being current sums of prediction errors $e_{3}\left(0\right)$ (outputs of NLMS2 stage), where $i_{k}$ is fixed and indicates the number of contexts chosen for coding of current pixel for the kth technique. $N_{\mathrm{map}}=[N_{{\mathrm{JPEG}-\mathrm{LS}}_{1}}\left(1,i_{1}\right),N_{{\mathrm{JPEG}-\mathrm{LS}}_{2}}\left(2,i_{2}\right),N_{{\mathrm{JPEG}-\mathrm{LS}}_{3}}\left(3,i_{3}\right),N_{{\mathrm{JPEG}-\mathrm{LS}}_{4}}\left(4,i_{4}\right),N_{{\mathrm{CALIC}}_{1}}\left(1,i_{1}\right),N_{{\mathrm{CALIC}}_{2}}\left(2,i_{2}\right),$ $N_{{\mathrm{CALIC}}_{3}}\left(3,i_{3}\right),\;N_{{\mathrm{CALIC}}_{4}}\left(4,i_{4}\right)]$ consist of current numbers of appearances of context number $i_{k}$. For j={9,10,11,12}, $C_{j}$ is the median $C_{\mathrm{Median}}\left(i_{k}\right)$ obtained as the middle value of a vector $V_{\mathrm{MED}}\left(i_{k}\right)$ containing up to 128 values of sorted prediction errors $e_{3}\left(0\right)$ for every context number $i_{k}$.

From a statistical point of view, careful averaging of uncertain measurements leads to improved measurement results. This is why the constant component is removed as in Formula (33) in this paper. At the same time, the approach reduces variations in histogram shapes, which may manifest in their strong asymmetry: for a given context i, there may be several maximum values $S_{\mathrm{map}}\left(j\right)$ not positioned at its center (Laplace distribution is symmetric and has one maximum) [39].

As in [38], four indices pointing at four context systems are applied to each method, which are described in the next subsection. A novelty is Formula (33) being adaptive:

(34) $$\alpha_{j}=\frac{\beta_{j}}{\sum_{i=1}^{12}\beta_{i}},$$

where:

(35) $$\beta_{j}={\bar{\omega }}_{j}\cdot \sqrt[{3}]{\frac{N_{\mathrm{map}}\left(j\right)}{\theta_{\mathrm{map}}\left(j\right)}},$$

where $\theta_{\mathrm{map}}\left(j\right)$ is the actual cumulated final square prediction error for index *j* $(\theta_{\mathrm{map}}=[\theta_{\mathrm{JPEG}-\mathrm{LS}}\left(i_{1}\right)$, $\theta_{\mathrm{JPEG}-\mathrm{LS}}\left(i_{2}\right),\theta_{\mathrm{JPEG}-\mathrm{LS}}\left(i_{3}\right),\theta_{\mathrm{JPEG}-\mathrm{LS}}\left(i_{4}\right),\theta_{\mathrm{CALIC}}\left(i_{1}\right),\theta_{\mathrm{CALIC}}\left(i_{2}\right),\theta_{\mathrm{CALIC}}\left(i_{3}\right),\theta_{\mathrm{CALIC}}\left(i_{4}\right),\theta_{\mathrm{Median}}\left(i_{1}\right),\theta_{\mathrm{Median}}\left(i_{2}\right),\theta_{\mathrm{Median}}\left(i_{3}\right),\theta_{\mathrm{Median}}\left(i_{4}\right)])$, which is updated as follows:

(36) $$\theta_{\mathrm{map}}^{\left(n+1\right)}\left(j\right)=\theta_{\mathrm{map}}^{\left(n\right)}\left(j\right)+{\left(P\left(0\right)-\dot{x}\left(0\right)\right)}^{2}.$$

The experimentally found weights were: ${\bar{\omega }}_{j}=\left\{0.275;0;0.4;0.15;0.2;0.3;0.1;0.35;0.2;0.2;0.325;0.2\right\}$; optimization of weights for a particular image is also possible. When a count $N_{\mathrm{map}}\left(j\right)$ reaches 128, then it is halved, similar to $S_{\mathrm{map}}\left(j\right)$. In this case, the denominator of (35) is scaled: $\theta_{\mathrm{map}}^{\left(n+1\right)}\left(j\right)=0.5\cdot \left(\theta_{\mathrm{map}}^{\left(n\right)}\left(j\right)+1000\right)$. In the case of median updating when halving the 128-element vector $V_{\mathrm{MED}}\left(i_{k}\right)$ (contains sorted prediction errors $e_{3}\left(0\right)$ for context number $i_{k}$), the greatest 32 and the smallest 32 are rejected.

### 4.2. Contexts for Correcting Prediction Error Bias

Three of four context definitions for the error bias correction methods are similar to those described in [38], but there are some improvements (Section 4.2.1–Section 4.2.3). The fourth approach is completely new (Section 4.2.4).

#### 4.2.1. Approach for *k* = 1

The context definitions in [11] use values of t samples surrounding the coded pixel P(0), and their mean. For t=8, the samples are P(1), P(2), P(3), P(4), P(5), P(6), GradNorth=2P(2)−P(6), and GradWest=2P(1)−P(5). The numbering is the same as in Figure 1. We take an 8-bit word and set each bit to 1 if the corresponding sample value is greater than the mean of the *t* samples, and 0 in the opposite case. As such, 1 of the 256 context numbers is defined. Additionally, we can quantize the pixel standard deviation to *q* values; the number of contexts grows to $q\cdot 2^{t}$. In this paper, t=8 and q=4 are used, which produces 1024 contexts $i_{1}$. The arithmetic mean is replaced by the expectation value ${\bar{y}}_{3}\left(0\right)=y_{1}\left(0\right)+y_{2}\left(0\right)+y_{3}\left(0\right)$. The variance (multiplied by 8) is:

(37) $${\hat{\sigma }}^{2}\left(0\right)={\left({\bar{y}}_{3}\left(0\right)-\mathrm{GradNorth}\right)}^{2}+{\left({\bar{y}}_{3}\left(0\right)-\mathrm{GradWest}\right)}^{2}+\sum_{i=1}^{6}{\left({\bar{y}}_{3}\left(0\right)-P\left(i\right)\right)}^{2}.$$

Quantization levels were 3 experimentally found thresholds for ${\hat{\sigma }}^{2}\left(0\right)$: 300, 2000, 8000. A similar idea was presented in [40].

#### 4.2.2. Approach for *k* = 2

The context-defining approach proposed here is similar to that used in JPEG-LS [12]. There are 6 contexts obtained from value ranges of neighbor pixel differences $d_{1}$, $d_{2}$, and $d_{3}$. Ranges are defined by difference signs and 2 thresholds, $q_{1}$ and $q_{2}$, which give $6^{3}=216$ contexts (a six-state quantizer with 5 thresholds $\left\{-q_{2},-q_{1},0,q_{1},q_{2}\right\}$). Differences $d_{j}$ are defined as follows: $d_{1}={\bar{y}}_{3}\left(0\right)-P\left(4\right)$, $d_{2}={\bar{y}}_{3}\left(0\right)-P\left(1\right)$, and $d_{3}={\bar{y}}_{3}\left(0\right)-P\left(2\right)$. In addition, a three-bit number $b_{0}b_{1}b_{2}$ is introduced; the first bit $b_{0}$ is set if condition $\left|P\left(1\right)-P\left(5\right)\right|>q_{3}$ is fulfilled as the $b_{1}$ sign of e(1) is used. Finally, $b_{2}=1$ if ${\bar{y}}_{3}\left(0\right)$ is greater than the average of pixels coded up to that moment. The experimentally found thresholds $q_{j}$ were {5, 18, 20}. This produces a total of $6^{3}\cdot 2^{3}=1728$ contexts $i_{2}$.

#### 4.2.3. Approach for *k* = 3

The third approach is based on a simplified vector quantization method [41]. Initial data analysis resulted in defining 16 vectors of centroids (each containing 3 pixels from the coded pixel neighborhood: V={P(1),P(2),P(4)}). Centroids are initialized as shown in Algorithm 3.
**Algorithm 3:** Centroids initialization.1 $\underset{j\in 0;15}{\mathrm{count}}\left(j\right)=1$ 2 $\underset{j\in 0;15,i\in 0;2}{\mathrm{centroid}}\left(j,i\right)=j\cdot 2^{4}$.

The simplified adaptive method for updating centroids is based on Euclidean distances of the current vector V={P(1),P(2),P(4)} from all 16 centroids. The 4-bit $\left(b_{0}b_{1}b_{2}b_{3}\right)$ label *l* of the closest centroid is concatenated with the other 6 bits (defined below) into a 10-bit context number $i_{2}$ (i.e., they have 1024 contexts $i_{3}$). The *l*th centroid is adapted as follows:

(38) $$\underset{i\in 0;2}{\mathrm{centroid}}\left(l,i\right):=\frac{\mathrm{count}(l)\cdot \mathrm{centroid}(l,i)+V(i)}{\mathrm{count}(l)+1}.$$

Then, the counter count(l) is increased by 1.

Next, two bits $b_{4}b_{5}$ of context number $i_{3}$ are decisions if the neighboring pixel P(1) and P(2) values are close enough to te expected coded pixel value ${\bar{y}}_{3}\left(0\right)$: $|{\bar{y}}_{3}\left(0\right)-P\left(j\right)|\geq 7$ and j={1,2}. Bits $b_{6}b_{7}$ are obtained from the comparison of ${\bar{y}}_{3}\left(0\right)$ with values of P(1) and P(2). If $P\left(j\right)<{\bar{y}}_{3}\left(0\right)$, then the bit is zero, j={1,2}.

Bit $b_{8}$ carries information if the current ${\bar{y}}_{3}\left(0\right)$ is greater than average of pixels coded up to that moment. Finally, bit $b_{9}$ is defined on the basis of the following majority formula: if at least 5 pixels from the set {P(3),P(4),P(5),P(6),P(7),P(8),P(9)} are greater than ${\bar{y}}_{3}\left(0\right)$, then the bit is zero.

#### 4.2.4. Approach for *k* = 4

This approach is completely new. We start with reorganizing values $\left\{P\left(1\right),P\left(2\right),{\bar{y}}_{3}\left(0\right)\right\}$ in ascending order; a new set $\left\{p_{r_{1}}\leq p_{r_{2}}\leq p_{r_{3}}\right\}$ is obtained. Then, (non-negative) differences are defined: $d_{1}=p_{r_{2}}-p_{r_{1}}$ and $d_{2}=p_{r_{3}}-p_{r_{2}}$. The differences are quantized using two thresholds: 5 and 18. There are 6 possible orderings of the set, and $3^{2}$ quantization levels for differences, which produeces 54 combinations. After adding a five-bit number $54\cdot 2^{5}=1728$, contexts $i_{4}$ are obtained. Bit zero of the number $\left(b_{0}\right)$ is set if $p_{r_{2}}$ (median value) is greater than average of pixels coded up to that moment. The next bit $\left(b_{1}\right)$ is the error e(1) sign. Two following bits $\left(b_{2}b_{3}\right)$ respond to two questions linked with pixel P(4): is it lower than ${\bar{y}}_{3}\left(0\right)$ and is substantially different than ${\bar{y}}_{3}\left(0\right)$, i.e., $\left|{\bar{y}}_{3}\left(0\right)-P\left(4\right)\right|\geq q_{3}$. The experimentally found $q_{3}$ value was 20. Finally, the fifth bit $\left(b_{4}\right)$ is one if $\left|P(1)-P(5)\right|\geq q_{3}$.

## 5. Context Adaptive Binary Arithmetic Coder (CABAC)

Among the practical applications of prediction errors entropy coders, the most effective are the adaptive arithmetic ones, although various variations of Huffman code are also used, including Rice and Golomb [21]. When measuring the characteristics of prediction errors, it is possible to determine the approximate type of distribution of the currently encoded value e(0) quite well. Based on this assumption, a contextual multi-value arithmetic encoder is usually constructed using not one but t probability distributions associated with context numbers from 0 to t−1. Theoretically, as the number of contexts increases, an improvement in compression efficiency is expected. Initially, we do not know the probability distributions; their shapes emerge when collecting incoming data. Hence, for large t, the problem of context dilution arises, i.e., for too long time shapes of some, probability distributions are not formed. We need a quick determination of their approximate target form. Therefore, a compromise should be found between the number of contexts and the speed of probability distribution adaptation. Most often, 8 [11], 16, or even 20 contexts are used [42]. An analysis of the influence of the number of contexts on bit average was presented [43].

Another approach consists of assuming an initial knowledge of the approximate probability distributions for each context. In this case, an arithmetic encode should be built that can determine which histogram is updated by the coded pixel.

This is a complex problem as it requires finding the best possible mathematical description of distributions matching a given image or class of images. This approach was analyzed based on Laplace, Gaussian, Student’s t, and Generalized Gaussian Distribution (GGD) [22]. The conclusions were followed [44], where a GGD was used. A similar solution using Student’s t distribution was also used in the TMW method [45], where a principle of blending probability distributions was introduced. However, further research showed that an important issue is the influence of the implemented prediction models on the type of distributions, where parameters describe the actual distributions of prediction errors sufficiently well [46].

A less-often-used approach consists of omitting the prediction stage and coding adaptively pixels instead of prediction errors [47].

Among the most effective codecs, the most often used is an adaptive version of a multivalue arithmetic coder with a reduced number of coding symbols [48] obtained using a non-linear quantization stage. A reduced number of symbols results in an increase in adaptation speed (there are fewer contexts). Even faster probability distribution adaptations can be achieved when using the binary version of the arithmetic encoder. Therefore, our proposed solution to the entropy coder is a completely new context adaptive binary arithmetic coder (CABAC). The absolute error value |e(0)| is coded by an adaptive Golomb code, then compressed by two arithmetic coders. Additionally, if the error is non-zero, then the third coder for sign compression is activated. This two-stage approach significantly improves coder adaptability to the quickly changing properties of image prediction error.

### 5.1. Short-Term Estimation of Probability Distribution

The presented rules for determining the context number are an extension of ideas from previous works [14,15,42,43]. Firstly, values $\omega_{1}$ and $\omega_{2}$ are computed:

(39) $$\begin{array}{cc} \omega_{1}=\max\{ & 2.3\cdot \left|e(1)\right|,2\cdot \left|e(2)\right|,1.6\cdot \left|e(4)\right|,0.95\cdot \left(\left|e(3)\right|+\left|e(4)\right|\right),\\ \; & 1.25\cdot \left(\left|e(5)\right|+\left|e(10)\right|\right),1.3\cdot \left|e(3)\right|,1.375\cdot \left(\left|e(1)\right|+\left|e(2)\right|\right),\\ \; & 0.4\cdot \left(\left|e(6)\right|+\left|e(7)\right|\right),0.4\cdot \left(\left|e(8)\right|+\left|e(9)\right|\right)\}, \end{array}$$

(40) $$\omega_{2}=\frac{1}{\delta }\sum_{j=1}^{m}{\bar{d}}_{j}\cdot |e(j)|,$$

where $\delta =\sum_{j=1}^{m}{\bar{d}}_{j}$ ad m=28. For ${\bar{d}}_{j}$, see the description of Formula (16). Next, the computed parameters are:

(41) $$\omega_{3}=\max\left\{2.1\cdot \omega_{1},10.2\cdot \omega_{2}\right\},$$

(42) $$\begin{array}{cc} \omega_{4}=\max\{ & \left|P(1)-P(3)\right|,\left|P(2)-P(4)\right|,1.1\cdot \left|P(1)-P(2)\right|,\\ \; & 0.7\cdot \left|P(2)-P(3)\right|,0.9\cdot \left|P(1)-P(4)\right|,0.9\cdot \left|P(3)-P(4)\right|\}, \end{array}$$

which are used for final calculation of ω:

(43) $$\omega =\omega_{3}+0.48\cdot \omega_{4}.$$

ω is quantized using t−1 thresholds $T_{h}\left(j\right)$, which for t=16, gives a 4-bit number $b_{\mathrm{medium}}$ of short-term probability distribution $T_{h}=\left\{3,7,12,18,24,31,39,49,59,72,90,115,140,170,210\right\}$.

### 5.2. Medium-Term Estimation of Probability Distribution

Golomb code is particularly well suited for coding data with a geometric distribution [49]. Parameter $m_{G}$ is chosen so that for *p*, the parameter of geometric probability distribution $p^{m_{G}}\approx 1/2$. The value of $m_{G}$ is searched for each coded |e(0)| among the 6 probability distributions of the form $G\left(i\right)=\left(1-p\right)p^{i}$. They are defined by $m_{G}$ values m={1,1,2,3,4,12}. The current p parameter is calculated as p=(K−1)/K, where $K=\omega_{2}$ for m=48 (40). Then, $m_{G}$ is evaluated [49,50]:

(44) $$m_{G}=\left\lceil -\frac{\log_{10}\left(1+p\right)}{\log_{10}p}\right\rceil .$$

According to a previous observation [51], $m_{G}\approx ln\left(2\right)K$. Then, the value of ln(2)K is quantized using thresholds {0.01,1.5,3.6,11.0,16.0}; the obtained index $b_{\mathrm{Golomb}}$ with values of {0,1,...,5} is used to select element of set m. The value $b_{\mathrm{Golomb}}$ is a part of a context number, and is constant when coding bits of Golomb word representing current |e(0)|. Golomb word consists of unary coded group number $u_{G}=\left\lfloor \left|e(0)\right|/m_{G}\right\rfloor$, and for $m_{G}>1$, the group element number $v_{G}=\left|e(0)\right|-u_{G}\cdot m_{G}$ (remainder of division by $m_{G}$) is coded using phased-in binary code, which is the variant of the Huffman code for sources with $m_{G}$ equally probable symbols [49]. Specifying the $k=\lceil \log_{2}m_{G}\rceil$ parameter means that in each group, the first $l=2^{k}-m_{G}$ elements $v_{G}$ are coded using k−1 bits, and the remaining m−l elements are coded as number $v_{G}+l$ using k bits [21]. The value |e(0)| is transformed into two bitstreams representing $u_{G}$ and $v_{G}$ in the Golomb code block (Figure 2).

### 5.3. Context Number Calculation

Binary sequences $u_{G}$ and $v_{G}$ are coded by separate binary arithmetic coders. The context number for $u_{G}$ is computed as follows:

(45) $$ctx_{u}=6\cdot \left(2^{4}\cdot b_{\mathrm{Golomb}}+b_{\mathrm{medium}}\right)+b_{\mathrm{unary}},$$

where $b_{\mathrm{unary}}$ is in the range {0,1,...,5} and denotes the number of currently coded $u_{G}$ bits (starting with the most significant one). If there are more than six bits, then $b_{\mathrm{unary}}=5$. Hence, there are 576 $ctx_{u}$ contexts. The number of contexts for $v_{G}$ is 192:

(46) $$ctx_{v}=2^{4}\cdot \left(2\cdot b_{\mathrm{Golomb}}+b_{\mathrm{phased}-\mathrm{in}}\right)+2^{3}\cdot b_{\omega }+2^{2}\cdot b_{\mathrm{binary}}+b_{\mathrm{unary}2},$$

where $b_{\mathrm{unary}2}=\min\left\{b_{\mathrm{unary}},3\right\}$ and $b_{\mathrm{binary}}$ is the most significant bit of $v_{G}$, $b_{\omega }$ is a one-bit number obtained by quantizing ω using threshold 49, and $b_{\mathrm{phased}-\mathrm{in}}$ is 0 for the first coded bit of $v_{G}$ and 1 otherwise. If $b_{\mathrm{phased}-\mathrm{in}}=0$, then $b_{\mathrm{binary}}=0$.

### 5.4. Long-Term Adaptation of Probability Distribution

Each context number is associated with counters of its zeros and ones: n(0) and n(1). The counter values cannot grow infinitely; when the sum n(0)+n(1) reaches a value $N_{\max}$, both counts are halved. For $u_{G}$, use $N_{\max}=2^{10}$, and the counters’ initial values are n(0)=n(1)=1. For $v_{G}$, the values are $N_{\max}=2^{11}$, and initially, n(0)=n(1)=16.

### 5.5. Sign Coding

Separate error e(0) sign coding is rather uncommon in binary arithmetic coders; hence, it is a particular feature of the coder presented in this paper. There are 32 contexts for sign e(0) coding (5-bit context number). Bits of the context number are: signs of neighbor errors sgn(e(1)) and sgn(e(2)) (Figure 1). Then, bit $b_{\omega }$; see the comment to (46). Finally, the last two bits are obtained from the four-state quantization of |e(0)| using thresholds {1,3,16}. Initial counts of zeros and ones are set to n(0)=n(1)=2. For the sign coder, $N_{\max}=2^{10}$.

## 6. Performance and Complexity Analysis of New Algorithms

### 6.1. Generalized Criterion for Minimizing the Bit Average of a Coder

In [26], they observed that minimization of the mean square error is not equivalent to minimization of the first-order entropy and the average bitrate of a coded image. This observation prompted us to search for the more accurate global static predictors from this point of view for each of 45 test images using the Minkowsky vector distance criterion [52]:

(47) $$L_{M}=(\sum_{n\in Q}{\left|x^{\left(n\right)}\left(0\right)-{\hat{x}}^{\left(n\right)}\left(0\right)\right|}^{M}{)}^{\frac{1}{M}},$$

where $x^{\left(n\right)}\left(0\right)$ and ${\hat{x}}^{\left(n\right)}\left(0\right)$ are reference and actual vector elements, e.g., actual samples and their estimates (1), respectively; Q is the data training area for predictive model learning. It appeared that best predictors were obtained for M values between 0.6 and 0.9 (compromise M=0.75). For comparison, in [26], the MMAE criterion meant that M=1; for M=2, the criterion was equivalent to MMSE. In [26], some improved results were obtained in comparison to MMSE; however, the improvements were not as evident for the final bitrate as for prediction error entropy. This was due to the extremely difficult task of joint optimization of modelling and entropy coding stages, at least for online methods. In the case of advanced offline techniques such as MRP [15], the optimization simply resulted in the iterative nature of such algorithms and in their high computational complexity (forward adaptation). Replacing MMSE by some version of the Minkovsky criterion led to an important increase in potentially enhanced method computational complexity, making it impractical.

**Figure 5:**
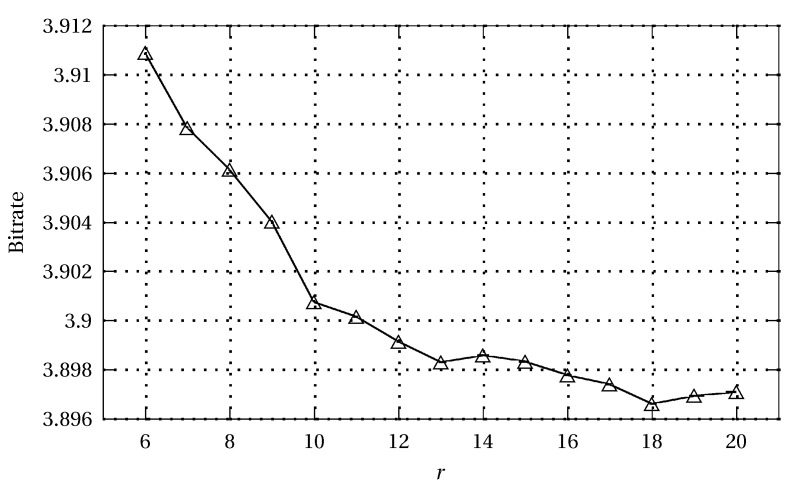
Dependence of the bit average on the LA-OLS prediction order (average for a set of 45 test images).

The results of [52] were so striking that we decided to continue experiments with the Minkovsky criterion. It appeared that the optimum value M can be strongly variable. We started with OLS prediction, followed by an adaptive arithmetic coder, and the value jumped from approximately 0.75 [52] to 1.3, partly due to the locality of the OLS predictors. After adding some kind of cumulated predictor error removal (compare with Section 4), the value increased to 1.5. The 3ST-OLS technique [31] has an additional NLMS+ stage (compare with Section 3.3), and for it, the optimal M is 1.7. Finally, for the proposed EM-WLS version, the best M value is 1.9 (Figure 5), and the gain in output data entropy with respect to MMSE criterion is very small and unlikely to be exploited in practice (of course, in the paper, we present the algorithm based on MMSE).

In summary, structures of the most effective data compacting algorithms seem to be collections of ad-hoc concepts that work, testing for how close to 2 the optimum *M* value is to the Minkovsky criterion for predictor optimization seems to validate these concepts, or not. For example, our findings highlight a non-obvious fact: multi-stage multimedia lossless coders are often more accurate than one-stage coders, in accordance with what is known about linear predictors. The added stages could even deteriorate the result; when data are scarce, combined total linear predictor length may be prolonged beyond limits given by length criteria, such as Akaike, MDL, etc. However, as in the examples above, the addition of a stage to a coder may push the optimum M value towards 2, and this seems to be the effect that matters.

**Figure 6:**
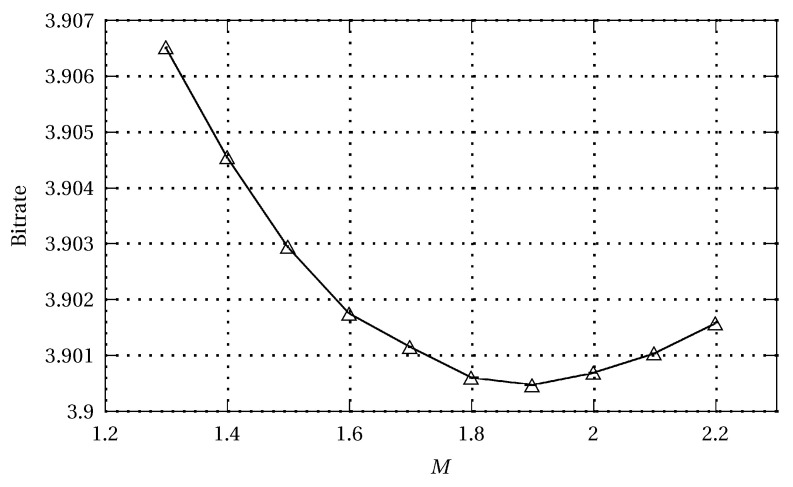
Relationship between the bit average and M, the parameter of the Minkovsky minimization criterion applied to the Main Predictor (20) in the EM-WLS method for a database of 45 test images.

In line with the findings presented here, the cascaded EM-WLS algorithm is close to optimal for the Minkovsky criterion (Figure 6), providing the advantages of an online approach (backward adaptation) and excellent performance (see Section 3.2).

### 6.2. Performance Analysis of Algorithm with LA-OLS in Main Predictor Stage

Testing of this method started with checking if each of the novel aspects of the method improved overall technique performance. Table 1 compares the average bitrates per pixel for the LA-OLS method (last column) with algorithm versions with an omitted feature. In Test 1, Formula (8) was implemented instead of (11); in Test 2, predictor coefficients were not weighted (12); in Test 3, there were no NLMS stages; and in Test 4, there was no error bias cancelling stage. As can be seen, the results for the full method were superior. The situation was similar when using EM-WLS as the Main Predictor.

Figure 5 shows the bit average for a set of 45 test images as a function of the LA-OLS predictor order. Table 2 presents results for LA-OLS predictor orders from 6 to 20, size of optimal training window size W, and execution times of the versions, times are for coding of Lennagray image on i5 3.4 GHz PC. As can be seen, the best parameters are obtained for r=18, and W=10. For this case total execution time is 5.86 s, which is very close to that for GLICBAWLS method [32]. Additional time needed for the realisation of NLMS and bias correction stages is quite big when these stages are appended to described in [53] CoBALP_ultra_ algorithm, its execution time extends from 2.07 s to 2.62 s.

### 6.3. Effects of Neighborhood Selection in Linear Prediction

**Figure 7:**
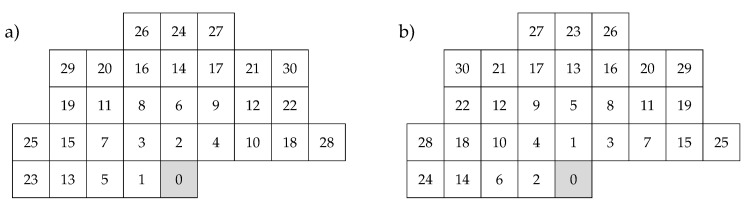
Two methods of numbering the pixels in the neighborhood of the currently coded pixel P(0).

The most accurate result is obtained when the closest possible neighborhood of the coded pixel (in terms of Euclidean distance) is selected, consisting of *r* pixels P(i) in (1), where *r* is the predictor order. This was confirmed by experiments. Notably, there are groups of pixels equidistant to the coded pixel P(0). For r≤30, these are pixels with numbers as shown in Figure 7a, where equidistant pixels are numbered clockwise: {1, 2}, {3, 4}, {5, 6}, {7, 8, 9, 10}, {11, 12}, {13, 14}, {15, 16, 17, 18}, {19, 20, 21, 22}, {23, 24}, {25, 26, 27, 28}, and {29, 30}. In the case of orders *r* belonging to the set {2, 4, 6, 10, 12, 14, 18, 22, 24, 28, 30}, pixel numbering is irrelevant, as long as we maintain the rule of the Euclidean distance minimization. Examples can be found in previous works for r=6 [54], for r=10 [30], for r=12 [33,55,56,57], and for r=18 [29].

A problem arises for other prediction orders, e.g., when r=5, we have to decide which pixel, P(5) or P(6), completes the set of four nearest pixels: P(1), P(2), P(3), and P(4). For example, in [58], the fifth neighbor was pixel P(5) (Figure 7a). The finding seems to be justified by the fact that we minimized the number of image rows, to which the procedure of calculating the predicted value must have access. This is important for optimizing execution time or resource usage for hardware solutions.

In many cases, the speed of calculations has high priority. In such cases, low orders of prediction are implemented as a compromise, and even some simplifications are introduced to reduce the complexity of Equation (8) calculation to close to that for the model of order r=5 while maintaining compression efficiency close to that offered by order r=6 [54].

Some solutions in the literature prefer the Manhattan ($l_{1}$ norm) over the Euclidean distance. For example, in [32], the neighborhood for distance $l_{1}\leq 3$ (r=12) was used, and in [44,46] distances were $l_{1}\leq 4$ (r=20) and $l_{1}\leq 5$ (r=30), respectively.

In our opinion, the best approach to the numbering of neighborhood pixels is to minimize the Euclidean distance. In the case of equidistant pixels, counterclockwise numbering should be used (Figure 7b). Our experiments confirmed the advantage of this approach over clockwise numbering. This advantage is most noticeable when using linear predictors of orders 3, 5, and 7. This is particularly important when using multiple prediction models of different orders together, as in Section 3.2.3.

An intuitive explanation is that, assuming the same level of correlation of equidistant neighbors from pixel P(0), the pixels on the right side of P(0) should be selected first. This is because the number of neighbors to the left of P(0) is predominant, for example, for r=14, there are seven of them; another three are located directly above the coded pixel, and on its right side, there are only four of them. The dominance of left-handed neighbors introduces some imbalance of information. Therefore, counterclockwise numbering, at least for some prediction orders, reduces this disproportion.

The authors of [26] used one step further in this direction by relaxing the Euclidean distance criterion for a model of order r=14. They decided to use only three rows, keeping the six neighbors to the left (two columns in each of three rows) equal to that of those to the right of the coded pixel P(0) (together with two pixels directly above it: four columns in each of two rows).

### 6.4. Performance Analysis of New Algorithms

Section 6.2 confirms the usefulness of all blocks in the proposed cascade model (see Figure 2) for total coder compression efficiency maximization. Additionally, the dependence is shown of coding time on parameters *r* and *W* when using LA-OLS as the Main Predictor. The highest efficiency was obtained for r=18 and W=10. For these settings, the encoding time of the Lennagrey image (512×512 pixels) is 5.86 s.

When EM-WLS was used in the first predictive block, the coding time increased significantly up to 107.4 s (decoding time was similar due to the same process for calculating predictions of coded pixels in the decoder). The time is not dependent on image content; it is only linearly proportional to the number of pixels in the encoded image.

Table 3 presents the time contributions of the EM-WLS cascade model coding stages to the whole coding time. It shows that the total time contribution of the last three stages is less than 1% (two last entries of Table 3). The same holds for total processing time of both NLMS blocks. Among the most complex operations is the determination of predictive model, requiring the solution of 22 matrix equations, which is performed twice due to the use of ridge-regression (Formulas (11) and (19)). This is performed using Cholesky decomposition, which requires 13.21% of the coding time. However, the most complex calculation is that of filling the R matrix and P vector (see Formulas (9) and (10)). The high computational complexity of this step is due to each coded pixel, for as much as W·(W+1)·r·(r+5), multiplications and additions should be performed despite symmetry of the R matrix. For r=24 and W=14, this necessitates 146,160 multiplication and addition operations.

Table 4, Table 5, Table 6 and Table 7 compare LA-OLS and EM-WLS performance to those of provided in the literature for other efficient lossless image coding techniques. Two sets of test images were used for this purpose [59,60]. The execution times of CALIC and JPEG-LS, JPEG2000, and BMF) are below one second. The methods in Table 4 are faster than EM-WLS, but perform worse: Blend-20 is three times faster, LA-OLS codes the Lennagray image in 5.86 s (Pentium i5 3.4 GHz), in contrast to 52.5 s for Vanilic WLS-D. The coding time using CoBALP_ultra2_, GLICBAWLS, 3ST-OLS, SWAP, and RALP is a few seconds.

Among methods with the most complex implementations, EM-WLS stands out with an execution time 107.4 s. This is still 3.9 times less than for MRP 0.5. GPR-BP, MRP-SSP, and TMW*^Lego^* are even more computationally complex. As can be seen, being less complex, EM-WLS is, on average, closer to optimum than these algorithms. However, decoders for MRP 0.5 and, hence, GPR-BP and MRP-SSP, are relatively time efficient due to the use of prediction with forward adaptation, whereas the EM-WLS decoder complexity is similar to that of a coder. EM-WLS files are on average 11.98% shorter than those of JPEG-LS.

## 7. Conclusions

In this work, different approaches to lossless compression of images, such as forward and backward adaptation, were analyzed. The paper contains some remarks on neighborhood selection in pixel prediction (Section 6.3) and notes on relationships between Minkovsky distance, final prediction error first-order entropy, and eventual coder average data rate (Section 6.4). The proposed EM-WLS algorithm construction was influenced by these observations. When compared to the best algorithms, on average, the proposed EM-WLS lossless image coding technique is currently the most efficient in terms of data compaction. The algorithm is less computationally complex than its main competitors. It is based on the AVE-WLS approach, being an expanded version of WLS, and has a cascade form, where the EM-WLS predictor is followed by a two-stage NLMS section, and by a final Context-Dependent Constant Component Removing stage. The new sophisticated binary context arithmetic coder is much less computationally complex than the preceding data modelling stage; hence, it can be used in other image compression methods. In the proposed universal cascade architecture (Figure 2), the Main Predictor module can be converted to an LA-OLS coder with lower implementation complexity (while maintaining high compression efficiency) due to the simpler prediction value calculation technique, as shown in Section 3.1.

## Figures and Tables

**Table 1:** Bitrate comparison of simplified and full LA-OLS versions.

Test	1	2	3	4	LA-OLS
Bitrate for 45 images	3.91414	3.90987	3.96234	3.97458	3.90510

**Table 2:** Performance of LA-OLS for a set of OLS orders (average for a set of 45 test images).

*r*	*W*	Bitrate for 45 Images	Execution Time (s)
6	6	3.91090	2.80
7	6	3.90785	2.94
8	6	3.90614	3.02
9	8	3.90404	3.30
10	8	3.90076	3.48
11	8	3.90017	3.65
12	8	3.89914	3.87
13	8	3.89832	4.08
14	10	3.89860	4.63
15	10	3.89834	4.90
16	10	3.89778	5.20
17	10	3.89741	5.52
18	10	3.89661	5.86
19	10	3.89695	6.21
20	10	3.89710	6.58

**Table 3:** Time contributions of algorithm steps to the total coding time.

Filling the R Matrix and P Vector	Solving Matrix Equations	Two NLMS Blocks	CDCCR	CABAC and Golomb Code
85.20%	13.21%	0.62%	0.85%	0.12%

**Table 4:** Bitrate comparison of some state-of-the-art algorithms for the first image database [59].

Images	JPEG-LS [12]	CALIC [11]	OLS [14]	GLICBAWLS [32]	CoBALP_ultra2_ [31,53]	Vanilc WLS-D [61]	3ST-OLS [31]
Balloon	2.889	2.78	2.690	2.640	2.673	2.626	2.580
Barb	4.690	4.31	3.939	3.916	3.881	3.815	3.832
Barb2	4.684	4.46	4.310	4.318	4.247	4.231	4.219
Board	3.674	3.51	3.388	3.392	3.339	3.332	3.296
Boats	3.930	3.78	3.638	3.628	3.591	3.589	3.544
Girl	3.922	3.72	3.576	3.565	3.523	3.523	3.471
Gold	4.475	4.35	4.273	4.276	4.232	4.229	4.208
Hotel	4.378	4.18	4.162	4.177	4.067	4.074	4.047
Zelda	3.884	3.69	3.549	3.537	3.568	3.501	3.504
**Bit average**	**4.058**	**3.864**	**3.725**	**3.717**	**3.665**	**3.658**	**3.633**

**Table 5:** Bitrate comparison of some state-of-the-art and proposed algorithms for the first image database [59].

Images	TMW^*Lego*^ [13]	LA-OLS	MRP 0.5 [15]	Multi-WLS [14]	Blend-20 [16]	AVE-WLS [62]	Extended Multi-WLS
Balloon	2.60	2.576	2.579	2.60	2.566	2.549	2.546
Barb	3.84	3.832	3.815	3.75	3.768	3.712	3.705
Barb2	4.24	4.214	4.216	4.18	4.175	4.134	4.126
Board	3.27	3.288	3.268	3.27	3.272	3.242	3.240
Boats	3.53	3.537	3.536	3.53	3.520	3.495	3.494
Girl	3.47	3.467	3.465	3.45	3.449	3.411	3.409
Gold	4.22	4.198	4.207	4.20	4.185	4.170	4.169
Hotel	4.01	4.040	4.026	4.01	4.007	3.979	3.977
Zelda	3.50	3.499	3.495	3.51	3.498	3.485	3.483
**Bit average**	**3.631**	**3.628**	**3.623**	**3.611**	**3.605**	**3.575**	**3.572**

**Table 6:** Bitrate comparison of some state-of-the-art algorithms for the second image database [60].

Images	JPEG2000 [47]	FLIF 0.3 [47]	WebP Lossless 0.6 [47]	SWAP [63]	RALP [57]	TMW [45]	GLICBAWLS [32]	PMO [47]
Airplane	4.013	3.794	3.894	3.58	3.71	3.601	3.668	3.632
Baboon	6.107	6.078	5.891	5.86	5.81	5.738	5.666	5.727
Balloon	3.031	2.856	2.925	2.49	2.55	2.649	2.640	2.673
Barb	4.600	4.500	4.547	4.12	4.12	4.084	3.916	3.997
Barb2	4.789	4.656	4.668	4.55	4.51	4.378	4.318	4.287
Camera	4.535	4.285	4.274	4.39	4.24	4.098	4.208	3.960
Couple256	3.915	3.677	3.703	3.75	3.63	3.446	3.543	3.415
Gold	4.603	4.518	4.464	4.30	4.32	4.266	4.276	4.476
Lennagrey	4.303	4.252	4.145	3.95	3.95	3.908	3.901	3.944
Peppers	4.629	4.595	4.495	4.25	4.27	4.251	4.246	4.267
**Bit average**	**4.453**	**4.321**	**4.301**	**4.124**	**4.111**	**4.042**	**4.038**	**4.038**

**Table 7:** Bitrate comparison of some state-of-the-art and new algorithms for the second image database [60].

Images	BMF [15]	Vanilc WLS-D [61]	xMRP [64]	MRP 0.5 [15]	LA-OLS	GPR-BP [17]	MRP-SSP [18]	Extended Multi-WLS
Airplane	3.602	3.575	3.590	3.591	3.568	3.451	3.536	3.547
Baboon	5.714	5.678	5.662	5.663	5.643	5.641	5.635	5.622
Balloon	2.649	2.626	2.613	2.579	2.576	2.544	2.548	2.546
Barb	3.959	3.815	3.817	3.815	3.832	3.821	3.764	3.705
Barb2	4.276	4.231	4.226	4.216	4.214	4.184	4.175	4.126
Camera	4.060	3.995	3.971	3.949	4.001	3.964	3.901	3.920
Couple256	3.448	3.459	3.389	3.388	3.414	3.339	3.323	3.345
Gold	4.238	4.229	4.216	4.207	4.198	4.178	4.173	4.169
Lennagrey	3.929	3.856	3.885	3.889	3.881	3.880	3.877	3.847
Peppers	4.241	4.187	4.208	4.199	4.153	4.170	4.163	4.101
**Bit average**	**4.012**	**3.965**	**3.958**	**3.950**	**3.948**	**3.917**	**3.910**	**3.893**
